# Lithium bidirectionally regulates depression- and mania-related brain functional alterations without worsening cognitive function in patients with bipolar disorder

**DOI:** 10.3389/fpsyt.2022.963005

**Published:** 2022-09-15

**Authors:** Chuanjun Zhuo, Guangdong Chen, Jiayue Chen, Hongjun Tian, Xiaoyan Ma, Qianchen Li, Lei Yang, Qiuyu Zhang, Ranli Li, Xueqin Song, Chunhai Huang

**Affiliations:** ^1^Key Laboratory of Real Time Tracing of Brain Circuits of Neurology and Psychiatry (RTBNP_Lab), Tianjin Fourth Center Hospital Affiliated to Tianjin Medical University, Tianjin Fourth Center Hospital, Tianjin, China; ^2^Department of Psychiatry, Wenzhou Seventh Peoples Hospital, Wenzhou, China; ^3^Key Laboratory of Psychiatric-Neuroimaging-Genetics Laboratory (PNGC_Lab), Tianjin Mental Health Center of Tianjin Medical University, Tianjin Anding Hospital, Tianjin, China; ^4^Department of Psychiatry, The First Affiliated Hospital of Zhengzhou University, Zhengzhou, China

**Keywords:** lithium, bipolar disorder, depression, mania, cerebral blood flow, functional magnetic resonance imaging, cognition

## Abstract

Lithium monotherapy has been proposed to have antidepressant and antimanic effects in patients with bipolar disorder (BP). However, so far, it is lack of evidence to support this proposition. The main aim of this study was to test the hypothesis that lithium bidirectionally regulates depression- and mania-related brain functional abnormalities in patients with BP. We also assessed the effects of lithium, alone and in combination with other pharmacological treatments, on patients' cognitive performance. We enrolled 149 drug-naïve patients with BP; 99 patients experiencing first depressive episodes were allocated randomly to four treatment groups [lithium (DP/Li), lithium with lamotrigine (LTG; DP/Li+LTG), LTG (DP/LTG), and valproate (VPA) with LTG (DP/VPA+LTG)], and 50 experiencing first hypo-manic episodes were allocated to two treatment groups (MA/Li and MA/VPA). For comparative analysis, 60 age-matched healthy individuals were also recruited. Whole-brain global and regional resting-state cerebral blood flow (rs-CBF) and cognitive alterations were examined before and after 12-week treatment. We have the following findings: DP/Li+LTG, and to a lesser extent DP/Li, alleviated the depression-related reduction in rs-CBF. MA/VPA and MA/Li reversed the mania-related elevation of rs-CBF completely and partially, respectively. Lithium alone improved cognitive performance during depressive and manic episodes; other tested treatments have no such effect or worsened cognitive ability. Our results showed that lithium bidirectionally regulates depression- and mania-associated brain functional abnormalities in patients with BP. Lithium monotherapy has a better antimanic effect than VPA, is superior to other tested treatments in improving cognition during the course of BP, and has satisfactory antidepressant effects in patients with BP.

## Introduction

Lithium, a well-known mood stabilizer, has long been used to treat mania in patients with bipolar disorder (BP), and as a synergist in the treatment of depression in these patients, especially those who have attempted suicide (1). These applications have been proven to be clinically effective (2–5), and based on data from many double-blind randomized controlled studies and other research, lithium is the gold-standard maintenance treatment for BP (6–15). More notably, Rybakowski modified the definition of mood stabilizer: ‘A drug that if used as mono-therapy: (1) act therapeutically in mania or/and in depression; (2) acts prophylactically against manic or/and depressive episodes as demonstrated in a trial of at least 1 year's duration and (3) does not worsen any therapeutic or prophylactic aspect of the illness outlined above.' According to this modified definition, lithium, valproate, and carbamazepine are classified as first-generation mood stabilizers, while atypical antipsychotics such as Clozapine, Quetiapine, Risperidone Lurasidone, Cariprazine, Asenapine, and Ziprasidone are classified as second-generation mood stabilizers (16, 17).

However, some debate exists regarding the use of the term “mood stabilizer;” whereas (16, 17) define lithium, divalproex, and second-generation antipsychotics as such (16, 17). Stephen stated that the U.S. Food and Drug Administration has not acknowledged the existence of mood stabilizers, and that the agents proposed by the APA [including second-generation antipsychotics and valproate (VPA)] have only anti-/hypomanic effects. Stephen (18) argues that “a true mood stabilizer should have bidirectional therapeutic effects, elevating depressive mood and suppressing manic symptoms.” More importantly, Stahl and Ketter argues that although lithium can be used as a synergist in the treatment of unipolar depression, including in patients who do not respond to antidepressant medications or follow electroconvulsive therapy; its therapeutic effectiveness for bipolar depression remains to be validated (18, 19). However, long-term lithium treatment can prevent major depression relapse, and this drug has better anti-suicide effects than do other mood stabilizers and second-generation antipsychotics (5, 20, 21). Lithium is also a clinically effective treatment for cognitive impairment, and potentially dementia (22–25). Barroilhet and Ghaemi (26) declared that lithium is the most effective pharmacological treatment in psychiatry, due mainly to its disease-modifying, rather than merely symptomatic, effects. Fountoulakis (27) and Fountoulakis et al. (28) demonstrated that lithium was effective for the treatment of acute mania occurring simultaneously with psychotic symptoms, and that its use in combination with other therapeutic agents was effective for acute bipolar depression. However, data on the effects of lithium on bipolar depression are inconsistent, and some researchers have concluded that lithium is ineffective for the treatment of this disorder (29) and long-term management of type I BP (30).

Strong evidence supports the neuroprotective effects of lithium treatment, which has led to its recommendation for dementia (31–34). In contrast, VPA and antipsychotics may worsen cognitive impairment. Xu et al. (35) reported that patients with BP were at greater risk of developing cognitive impairment as a disease symptom or treatment consequence. Using a C57BL/6 mouse model and micro-imaging, Nguyen et al. (36) demonstrated that lithium rescued cognitive impairment by normalizing cellular and molecular abnormalities in the prefrontal lobe, parietal lobe, and hippocampus. Furthermore, lithium has been shown to improve depressive and cognitive deficits in rodent models of cortical neuroinflammation and tauopathy (37). Burdick et al. (38) found that lithium improved the neurocognition of 262 patients with BP. An international multicenter magnetic resonance imaging (MRI) study revealed that lithium prevented gray-matter atrophy, especially in regions associated with mood processing, in patients with BP (39). However, these studies examined static states, rather than dynamic switching between depression and mania, as occurs in BP. Recently, McGhee et al. (40) used DNAzyme-based lithium-selective imaging to reveal increased lithium ion accumulation in differentiated neurons from patients with BP relative to healthy controls. However, the effect of lithium on cognitive ability, and the underlying mechanisms, in patients with BP remain to be characterized.

In this study, we used functional magnetic resonance imaging (fMRI) and measurements of resting-state cerebral blood flow (rs-CBF) (41–43) to test the hypothesis that lithium bidirectionally regulates depression- and mania-related brain functional abnormalities, thereby exerting bidirectional therapeutic effects, in drug-naïve patients with BP. We also assessed the effects of lithium, alone and in combination with other pharmacological treatments, on cognition during depressive and manic episodes in these patients. The findings may advance our understanding of the role of lithium in the treatment of BP, ultimately improving care for patients with this disorder.

## Methods

### Participants

Drug-naïve male patients aged 18–45 years with BP and family histories of this disorder in first-degree relatives were invited to participate in this study. All participants attended Tianjin Fourth Center Hospital. The hospital's ethics committee approved this study (No. 20191109KL), and all participants provided written informed consent prior to study participation. Sociodemographic and clinical data were collected from all individuals enrolled in the study. We included only male patients due to the possible influences of the menstrual cycle and status on the course of mood disorders after baseline assessment was performed to determine eligibility for study participation. The patients were stratified into those experiencing their first depressive and hypomanic episodes. For patients experiencing their first depressive episodes, the following inclusion criteria were used: (1) symptoms of depression fulfilling the Diagnostic and Statistical Manual of Mental Disorders, fourth edition (DSM-IV) criteria for a depressive episode, as diagnosed by psychiatrists using the structured clinical interview for DSM disorders (SCID); (2) had a hypo-manic episode history but did not accept any treatment (the hypo-manic was defined also according to DSM-IV), (3) no prior treatment with antidepressants or psychiatric therapeutic agents; and (4) full insight about the symptoms of mood disturbance, as determined using the Birchwood Insight Scale (BIS) and Beck Cognitive Insight Scale (BCIS). The inclusion criteria for patients experiencing their first manic episodes were: (1) current experience of a hypo-manic episode with symptoms fulfilling the DSM-IV criteria for such episodes, as determined by psychiatrists using the SCID; (2) first hypo-manic episode secondary to the first depression episode (defined according to DSM-IV) without a history of antidepressant treatment; (3) no prior treatment with psychiatric therapeutic agents; and (4) full insight about the symptoms of mood disturbance, as determined with the BIS and BCIS” (44). The inclusion criteria for patients experiencing their first hypomanic episodes were: (1) current experience of a hypomanic episode with symptoms fulfilling the DSM-IV criteria for such episodes, as determined by psychiatrists using the SCID; (2) history of an untreated depressive episode, defined according to the DSM-IV; (3) no prior treatment with psychiatric therapeutic agents; and (4) full insight about the symptoms of mood disturbance, as determined with the BIS and BCIS. Exclusion criteria were: (1) history of a mental disorder; (2) psychiatric illness caused by traumatic brain injury; (3) mental health issues in relation to physical illness; (4) personality disorder; (5) previous or current substance abuse; and (6) substantial loss of normal memory, as determined with the fourth edition of the Chinese version of the Wechsler Memory Scale (45). For comparative analysis, 60 age-matched healthy male individuals were also recruited; individuals with any documented psychiatric or psychological history were excluded.

### Assessment of mood episode symptoms and cognition

Among patients with BP, the 17-item Hamilton Depression Rating Scale (HAMD-17) (46) was used to assess the severity of depressive episode symptoms and the Bech-Rafaelsen Mania Rating Scale (BRMS) (47) was used to evaluate the severity of manic episode symptoms. The assessment of mood episode symptoms was performed before and after 4, 8, or 12 weeks of treatment. The MATRICS Consensus Cognitive Battery (MCCB) (48–50) was used to assess cognition before and upon completion of 12-week treatment.

### Treatments

In total, six treatments were administered for a 12-week period. Patients with BP experiencing depressive episodes were randomly allocated to four treatment groups: (1) lithium monotherapy (DP/Li), (2) lithium and lamotrigine (LTG; DP/Li+LTG), (3) LTG monotherapy (DP/LTG), and (4) VPA and LTG (DP/LTG+VPA). Patients with BP experiencing hypomanic episodes were randomly allocated to treatment with lithium monotherapy (MA/Li) or VPA monotherapy (MA/VPA). All lithium doses were 600 mg per day. Blood lithium concentrations were monitored according to the BP treatment guidelines.

### fMRI data acquisition

fMRI examinations were performed with a 3.0-Tesla system (Discovery MR750; General Electric, Milwaukee, WI, USA). Foam padding was used to minimize participants' head motion. Perfusion imaging was conducted during a pseudo-continuous arterial spin labeling (ASL) sequence with three-dimensional (3D) fast-spin-echo acquisition and background suppression [repetition time (TR) = 4886 ms, echo time (TE) = 10.5 ms, post-label delay = 2025 ms, eight-arm spiral readout with 512 sample points, flip angle = 111°, field of view (FOV) = 240 × 240 mm, reconstruction matrix = 128 × 128, slice thickness = 4 mm, no gap, 40 axial slices, number of excitations = 3, 1.9 × 1.9-mm in-plane resolution]. The total rs-ASL acquisition time was 4 min 44 s. During the MRI procedures, the participants were instructed to relax, keep their eyes closed, move as little as possible, and think of nothing without falling asleep. All acquired images were inspected visually to ensure that only qualified images were included in subsequent analyses (51).

### CBF mapping

rs-CBF, measured during ASL imaging, was quantified using a single compartment model after the subtraction of labeling images from control images. rs-CBF maps were subsequently derived from ASL difference images and proton density–weighted reference images. Statistical Parametric Mapping version 8 (SPM8) (Mathworks Inc, Sherborn, MA, USA) was used to co-register the rs-CBF images of the 60 healthy controls to a positron emission tomography perfusion template in the Montreal Neurological Institute (MNI) space using non-linear transformation. The standard rs-CBF template of the MNI was defined as the mean of these co-registered images. rs-CBF images of all patients were co-registered to this template and resampled to a voxel size of 2 × 2 × 2 mm. Non-brain tissue was removed from each co-registered rs-CBF map, and spatial smoothing was performed with a Gaussian kernel of 8 × 8 × 8 mm full width at half maximum. We normalized the rs-CBF for each voxel by dividing it by the mean whole-brain rs-CBF (51–53).

### Statistical analysis

Differences in rs-CBF among groups were examined with voxel-wise one-way analysis of covariance (ANCOVA) with age and sex serving as covariates, followed by *post-hoc* intergroup comparisons performed within a mask showing rs-CBF differences from the ANCOVA. The family-wise error (FWE) method was applied to correct for multiple comparisons. *P* < 0.05 was set as the significance threshold.

To evaluate the relationships of the rs-CBF to the total HAMD-17 and BRMS scores, we conducted a voxel-wise multiple regression analysis of data from the four DP and two Mania treatment groups. We targeted regions showing significant differences in rs-CBF from the healthy control groups. Pretreatment rs-CBF values were adjusted for patient characteristics, including age, illness duration, and severity of depressive episode symptoms in the DP groups or manic episode symptoms in the mania groups. The FWE method was used to correct for multiple comparisons (52, 53).

## Results

### Participants

We acquired fMRI data from a total of 209 participants: 60 healthy individuals, 99 patients with BP experiencing their first depressive episodes (25 in the DP/Li group, 28 in the DP/Li+LTG group, 22 in the DP/LTG group, and 24 in the DP/VPA+LTG group), and 50 patients with BP experiencing their first hypomanic episodes (26 in the MA/Li group and 24 in the MA/VPA group). Participants' sociodemographic and clinical characteristics are summarized in Table 1.

**Table 1:** Sociodemographic and clinical characteristics of patients with BP and healthy controls.

**Drug-naïve BP patients in the first episode of depression which had a hypo-manic episode history**						
**Variable**	**Health controls**	**DP/Li**	**DP/Li+LTG**	**DP/LTG**	**DP/LTG+VPA**	**ANOVA**
Age	24.50 ± 2.69	22.40 ± 2.17	37.70 ± 2.43	28.33 ± 1.78	35.17 ± 3.18	<0.0001
Education level	16.42 ± 4.30 (years)	15.58 ± 2.89 (years)	16.58 ± 6.75 (years)	16.00 ± 7.57 (years)	14.28 ± 5.26 (years)	0.089
Illness Duration	NA	6.52 ± 1.35 (months)	8.20 ± 2.14 (months)	4.99 ± 1.87 (months)	7.96 ± 0.51 (months)	<0.0001
HAMD (Scores) Before treatment	NA	36.49 ± 2.13	37.50 ± 3.44	38.35 ± 4.07	36.77 ± 1.99	0.049
HAMD (Scores) After treatment	NA	13.22 ± 2.01	9.87 ± 0.95	10.28 ± 3.13	14.99 ± 2.75	0.027
**MCCB before treatment**						
Speed procession	44.30 ± 5.45	35.14 ± 4.25	39.07 ± 2.30	39.22 ± 2.27	38.35 ± 1.69	0.033# 0.799*
Attention vigilance	46.47 ± 6.25	36.55 ± 3.12	37.53 ± 3.02	36.99 ± 5. 16	37.02 ± 3.40	0.017# 0.880*
Working memory	46.65 ± 4.30	36.57 ± 2.39	38.22 ± 3.20	37.03 ± 0.97	37.50 ± 1.67	0.007# 0.923*
Verbal learning	47.20 ± 2.77	37.68 ± 2.24	38.45 ± 2.87	38.99 ± 0.99	40.08 ± 3.99	0.012# 0.885*
Visual learning	48.56 ± 6.23	36.09 ± 3.12	33.03 ± 1.88	32.75 ± 2.14	33.02 ± 1…67	0.003# 1.997*
Reasoning	47.25 ± 2.58	39.24 ± 3.38	39.97 ± 3.69	38.55 ± 5.42	40.11 ± 2.77	0.015# 0.771*
Social recognition	48.99 ± 5.84	47.15 ± 3.69	43.72 ± 1.78	42.05 ± 3.56	43.10 ± 4.11	0.014# 0.920*
Composite	44.03 ± 2.70	31.26 ± 3.09	30.99 ± 3.76	31.00 ± 4.44	32.17 ± 1.32	0.025# 0.777*
**MCCB after treatment**						
Speed procession	44.30 ± 5.45	33.74 ± 5.10	33.01 ± 1.85	28.37 ± 2.51	26.95 ± 2.00	0.002# 0.021*
Attention vigilance	46.47 ± 6.25	36.00 ± 1.11	32.00 ± 1.11	29.97 ± 1.29	25.44 ± 1.05	0.009# 0.022*
Working memory	46.65 ± 4.30	36.24 ± 1.35	33.13 ± 1.93	29.43 ± 1.14	25.38 ± 1.19	0.011# 0.032*
Verbal learning	47.20 ± 2.77	36.07 ± 1.85	34.10 ± 1.58	31.63 ± 1.27	26.04 ± 2.25	0.019# 0.034*
Visual learning	48.56 ± 6.23	39.11 ± 1.00	28.94 ± 2.20	26.00 ± 1.57	22.78 ± 1.89	0.004# 0.029*
Reasoning	47.25 ± 2.58	40.00 ± 1.52	36.02 ± 1.75	30.72 ± 1.87	26.15 ± 1.80	0.003# 0.015*
Social recognition	48.99 ± 5.84	49.00 ± 0.79	39.67 ± 1.00	33.00 ± 1.27	32.77 ± 0.53	0.014# 0.032*
Composite	44.03 ± 2.70	30.99 ± 0.28	28.44 ± 1.58	25.33 ± 1.26	21.22 ± 0.99	0.011# 0.039*
**Drug naïve BP patients in the first episode of hypo-mania secondary to the first depressive episode**						
**Variable**	**HCs**	**MA/Li**	**MA/VPA**			
Education level	16.42 ± 4.30 (years)	16.85 ± 4.57 (years)	16.07 ± 3.11 (years)			0.762
Illness duration	NA	10.20 ± 4.24 (months)	8.21 ± 1.0.10 (months)			0.035
BRMS scores before treatment	NA	15.87 ± 2.35	16.25 ± 1.99			0.962
BRMS scores after treatment	NA	2.47 ± 1.66	2.30 ± 0.87			0.711
**MCCB before treatment**						
Speed procession	44.30 ± 5.45	36.24 ± 1.71	36.40 ± 2.21			0.033# 0.959*
Attention vigilance	46.47 ± 6.25	35.00 ± 0.84	36.12 ± 2.15			0.027# 0.937*
Working memory	46.65 ± 4.30	37.25 ± 1.03	36.93 ± 1.75			0.035# 0.966*
Verbal learning	47.20 ± 2.77	37.55 ± 1.70	36.99 ± 1.09			0.018# 0.994*
Visual learning	48.56 ± 6.23	35.17 ± 0.85	36.22 ± 0.77			0.025# 0.888*
Reasoning	47.25 ± 2.58	42.15 ± 0.69	42.00 ± 2.02			0.042# 0.971*
Social recognition	48.99 ± 5.84	43.05 ± 1.22	44.03 ± 2.13			0.031# 0.988*
Composite	44.03 ± 2.70	31.00 ± 0.87	32.15 ± 2.27			0.017# 0.903*
**MCCB after treatment**						
Speed procession	44.30 ± 5.45	26.79 ± 1.80	21.13 ± 2.25			0.003# 0.015*
Attention vigilance	46.47 ± 6.25	24.89 ± 2.34	18.36 ± 1.89			<0.001# 0.005*
Working memory	46.65 ± 4.30	28.00 ± 1.88	19.44 ± 1.75			<0.001# 0.006*
Verbal learning	47.20 ± 2.77	27.21 ± 2.10	19.84 ± 2.55			<0.001# 0.004*
Visual learning	48.56 ± 6.23	25.42 ± 0.99	17.01 ± 1.35			<0.001# 0.001*
Reasoning	47.25 ± 2.58	33.15 ± 0.69	23.00 ± 2.02			0.010# 0.015*
Social recognition	48.99 ± 5.84	31.97 ± 1.50	26.00 ± 250			0.003# 0.019*
Composite	44.03 ± 2.70	21.33 ± 0.29	16.66.15 ± 1.35			<0.001# 0.011*

^#^Comparison including HCs; *Comparison only among four treatment groups.

### rs-CBF alterations in the DP groups

**Figure 1:**
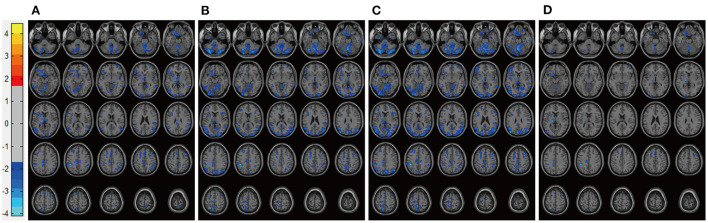
Representative images of pretreatment rs-CBF in patients with BP experiencing their first depressive episodes compared with the rs-CBF of healthy controls. **(A)** Before lithium monotherapy, **(B)** before LTG monotherapy, **(C)** before treatment with lithium and LTG, **(D)** before treatment with VPA and LTG. rs-CBF, resting-state cerebral blood flow; BP, bipolar disorder; LTG, lamotrigine; VPA, valproate.

**Figure 2:**
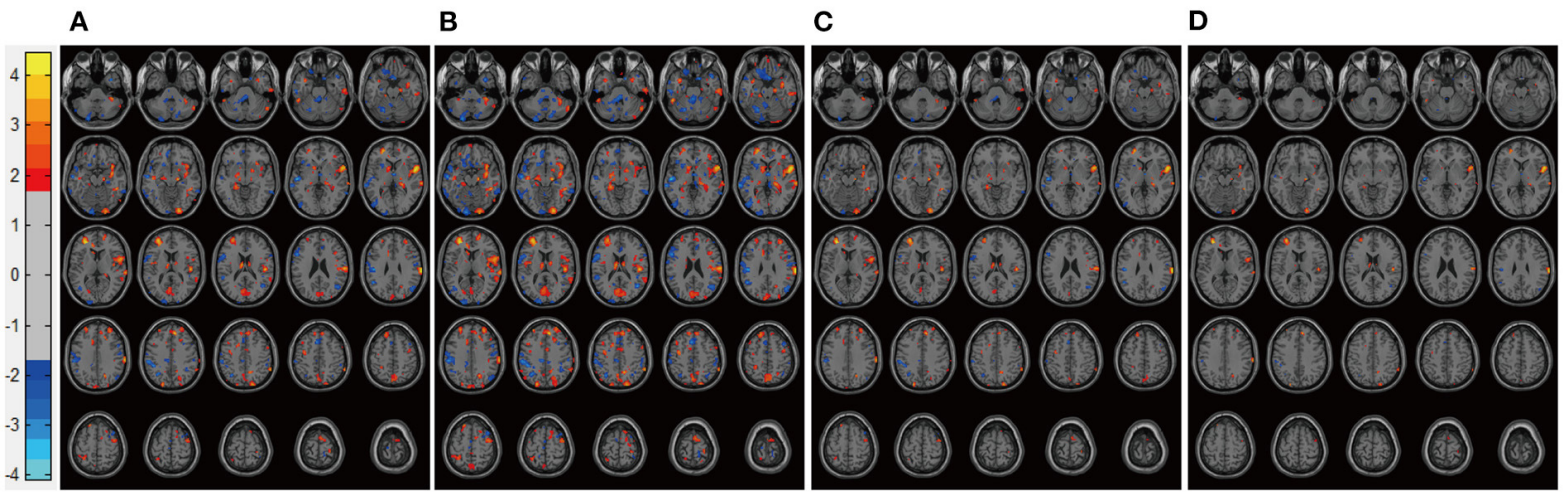
Representative images of post-treatment rs-CBF in patients with BP experiencing their first depressive episodes. **(A)** DP/Li **(B)** DP/LTG, **(C)** DP/Li+LTG, and **(D)** DP/VPA+LTG. rs-CBF, resting-state cerebral blood flow; BP, bipolar disorder; DP, patients with BP experiencing their first depressive episodes; DP/Li, treatment with lithium; DP/LTG treatment with lamotrigine; DP/Li+LTG, treatment with lithium plus lamotrigine; DP/VPA+LTG, treatment with valproate plus lamotrigine.

After adjustment for patient characteristics, patients in the DP groups had significantly lower pretreatment rs-CBF values than did healthy controls, mainly in Brodmann areas 11, 25, 39, 40, and 47 (Figure 1, Table 2). In each DP group, the global rs-CBF was increased after treatment relative to baseline (Figure 2, Table 2). This increase was greater in the DP/Li+LTG group than in the DP/Li group, and greater in the DP/LI+LTG group than in the DP/VPA+LTG group. Although distinct regional rs-CBF alteration patterns were observed in the four treatment groups, the main regions with increased rs-CBF were Brodmann areas 11, 25, 39, 40, and 47.

**Table 2:** rs-CBF alterations before and after treatment.

**rCBF alterations before treatment in the depressive-phase of BP**					
**Variable**	**DP/Li**	**DP/Li+LTG**	**DP/LTG**	**DP/LTG+VPA**	**ANOVA**
**Mainly brain regions (voxel numbers)**					
Cerebellum	1024.55 ± 258.99	2934.45 ± 699.12	4588.70 ± 1223.54	889.35 ± 102.96	<0.001# <0.001*
Occipital lobe	933.15 ± 103.60	3059.11 ± 1325.00	2487.77 ± 455.32	507.40 ± 45.80	<0.001# <0.001*
Frontal lobe	235.55 ± 70.00	800.03 ± 109.25	2213.79 ± 555.00	98.83 ± 28.56	<0.001# <0.001*
Parietal lobe	356.20 ± 89.90	999.74 ± 154.44	1023.79 ± 455.73	21.12 ± 6.98	<0.001# <0.001*
**rCBF alterations after treatment in the depressive-phase of BP**					
**Mainly brain regions (voxel numbers)**					
**Variable**	**DP/Li**	**DP/Li**+**LTG**	**DP/LTG**	**DP/LTG**+**VPA**	**ANOVA**
Cerebellum	333.45 ± 54.50	439.63 ± 107.99	111.22 ± 24.33	72.90 ± 15.55	<0.001# <0.001*
Occipital lobe	503.20 ± 70.35	1445.36 ± 270.99	302.20 ± 32.11	97.88 ± 10.89	<0.001# <0.001*
Parietal lobe	998.00 ± 130.27	1358.39 ± 223.14	605.47 ± 124.30	366.65 ± 99.35	<0.00# <0.001*
Frontal lobe	1313.46 ± 185.40	2549.68 ± 577.43	599.66 ± 74.88	103.37 ± 35.46	<0.001# <0.001*
Temporal lobe	144.35 ± 33.77	946.60 ± 122.54	203.00 ± 56.69	58.47 ± 9.85	<0.001# <0.001*
Basal ganglia	325.20 ± 74.58	877.60 ± 175.59	109.57 ± 34.22	11.83 ± 2.48	<0.001# <0.001*
**rCBF alterations pretreatment in the hypomanic-phase of BP**					
**Mainly brain regions (voxel numbers)**					
**Variable**	MA/Li	MA/VPA			
Cerebellum	479.32 ± 98.60	500.50 ± 110.45			0.075
Occipital lobe	894.77 ± 231.25	951.28 ± 253.36			0.025
Parietal lobe	429.64 ± 58.97	701.10 ± 96.25			<0.001
Frontal lobe	1541.66 ± 256.99	1815.45 ± 345.63			0.007
Temporal lobe	357.77 ± 77.55	380.22 ± 103.24			0.051
Basal ganglia	295.54 ± 56.37	275.87 ± 89.58			0.056
**rCBF alterations post-treatment in the hypomanic-phase of BP**					
**Mainly brain regions (voxel numbers)**					
**Variable**	MA/Li	MA/VPA			
Cerebellum	0	34.88 ± 6.14			
Occipital lobe	58.35 ± 11.24	0			
Parietal lobe	25.40 ± 4.69	0			
Frontal lobe	111.20 ± 41.23	0			
Temporal lobe	84.40 ± 12.65	0			
Basal ganglia	0	18.54 ± 2.0.10			

### rs-CBF alterations in the hypomania groups

**Figure 3:**
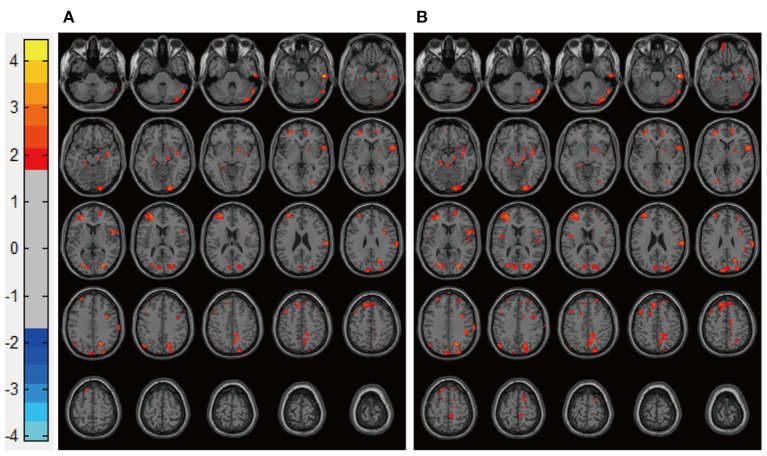
Representative images of pretreatment rs-CBF in patients with BP experiencing their first manic episodes compared with the rs-CBF of healthy controls. **(A)** Before lithium monotherapy, **(B)** before VPA monotherapy. rs-CBF, resting-state cerebral blood flow; BP, bipolar disorder; VPA, valproate.

**Figure 4:**
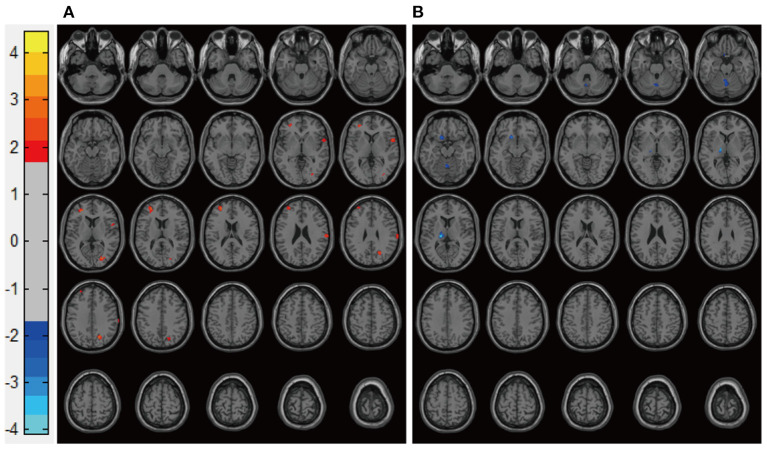
Representative images of post-treatment rs-CBF in patients with BP experiencing their first manic episodes. **(A)** MA/Li, **(B)** MA/VPA. rs-CBF, resting-state cerebral blood flow; BP, bipolar disorder; MA, patients with BP experiencing their first manic episodes; MA/Li, treatment with lithium; MA/VPA, treatment with valproate.

Patients in the Mania groups had higher pretreatment rs-CBF values than did healthy controls, affecting Brodmann areas 10, 13, 22, 43, 44, and 47 (Figure 3, Table 2). Post-treatment global rs-CBF was reduced in both treatment groups; the increases were fully and partially reversed in the MA/VPA and MA/Li groups, respectively (Figure 4, Table 2).

### Treatment effects on patients' symptoms

**Figure 5:**
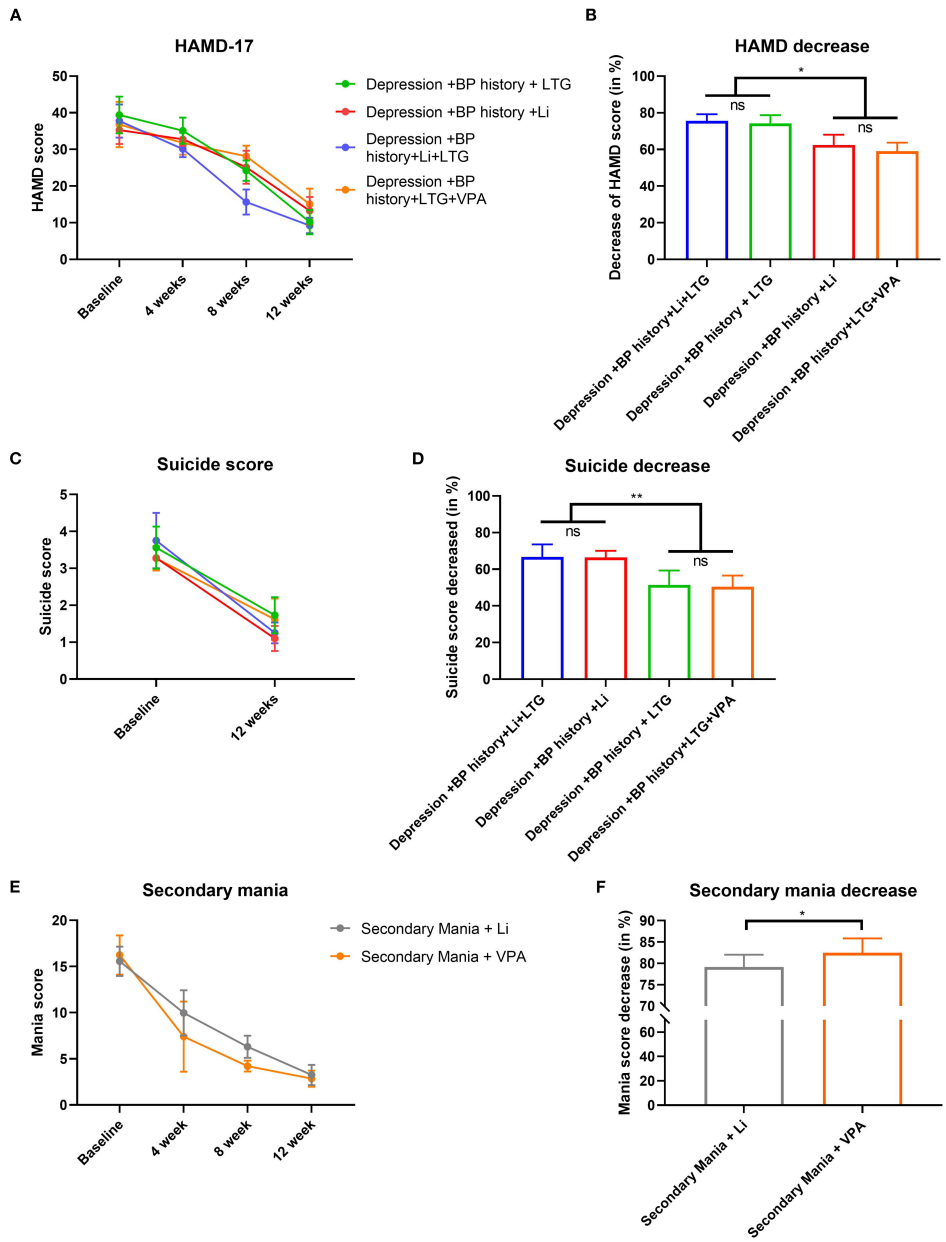
Effects treatments on HAMD-17, suicidality or BRMS scores of patients with BP. For patients experiencing their first depressive episodes, **(A)** HAMD-17 scores before treatment and at 4, 8, and 12 weeks; **(B)** reductions in HAMD-17 scores; **(C)** suicidality scores before and after 12 weeks of treatment; **(D)** reductions in suicidality scores. For patients experiencing their first manic episodes, **(E)** BRMS scores before treatment and at 4, 8, and 12 weeks and **(F)** reductions in BRMS scores. HAMD-17, the 17-item Hamilton Depression Rating Scale; BRMS, the Bech-Rafaelsen Mania Rating Scale; BP, bipolar disorder. *p < 0.001 and **p < 0.0001.

Depression symptoms were markedly improved after treatment in all four DP groups: the HAMD-17 score had decreased significantly, especially in the DP/LTG and DP/Li+LTG groups (with no significant difference between these groups; Figures 5A,B). Post-treatment suicidality scores were also reduced significantly in all four DP groups, with significantly greater reduction observed in the DP/Li+LTG and DP/Li groups than in the DP/LTG and DP/VPA+LTG groups (Figures 5C,D). BRMS scores had decreased significantly in both mania groups after treatment, with a greater reduction observed in the MA/VPA group than in the MA/Li group (Figures 5E,F).

### Treatment effects on patients' cognitive performance

**Figure 6:**
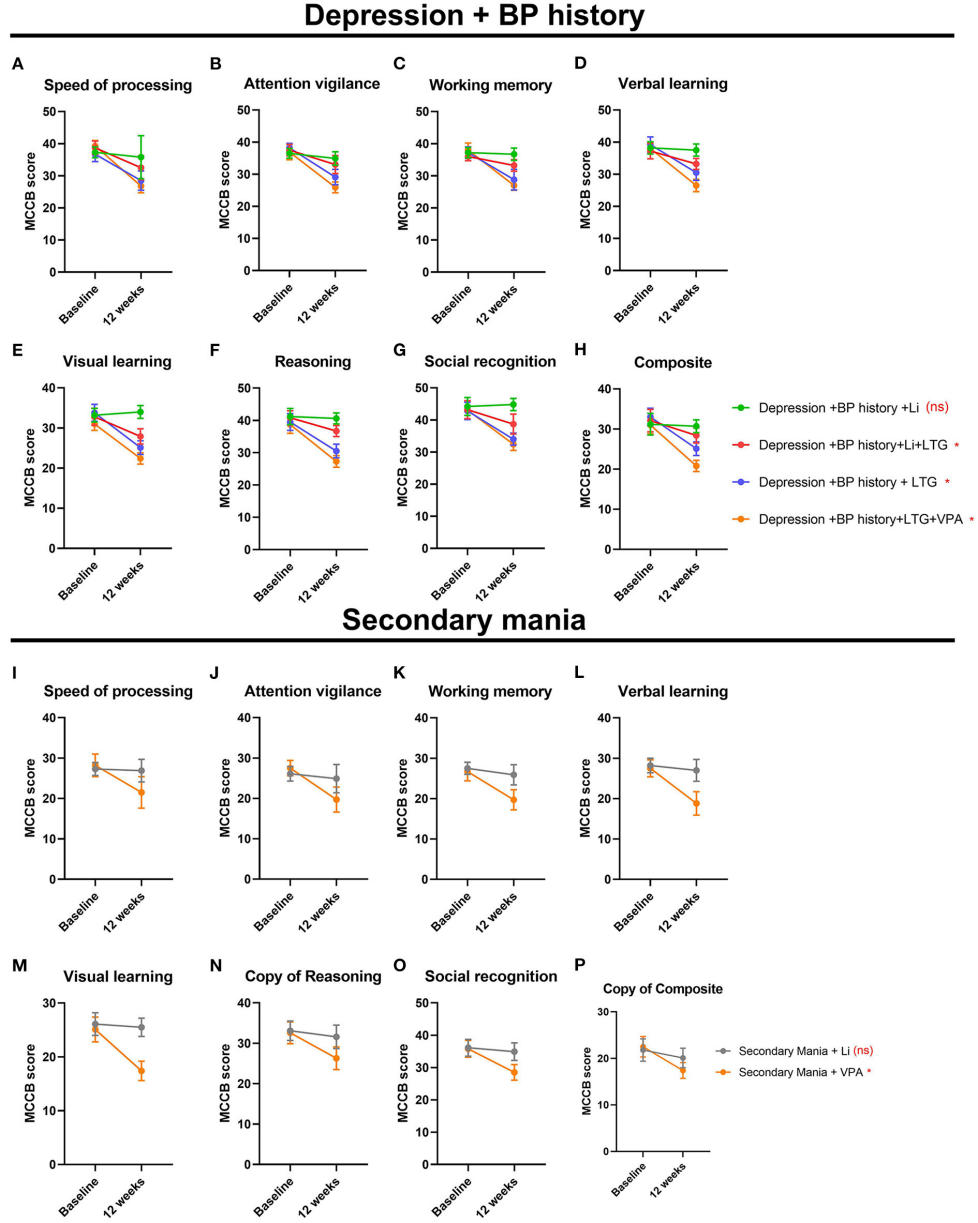
Treatment effects on the cognitive performance of patients with BP. For patients experiencing their first depressive and manic episodes, respectively **(A,I)** processing speed, **(B,J)** attention vigilance, **(C,K)** working memory, **(D,L)** verbal learning, **(E,M)** visual learning, **(F,N)** reasoning, **(G,O)** social recognition, and **(H,P)** composite MCCB scores before and after 12 weeks of treatment. BP, bipolar disorder; MCCB, the MATRICS Consensus Cognitive Battery.

Our data demonstrated that the cognitive performance was differently in different treatment groups both in the depressive-phase and in the hypomanic phase. In the DP/Li group, the alterations of the elements of the MCCB before and after treatment demonstrated none significantly differences. For example, speed procession scores before treatment was 35.14 ± 4.25, after treatment, was 33.74 ± 5.10; repeated ANOVA analysis did not demonstrate significant differences. Similar, attention vigilance (36.55 ± 3.12_beforetreatment_ vs. 36.00 ± 1.11_aftertreatment_, *P* = 0.063); working memory (36.57 ± 2.39_beforetreatment_ vs. 36.24 ± 1.35 _aftertreatment_, *P* = 0.069); verbal learning (37.68 ± 2.24_beforetreatment_ vs. 36.07 ± 1.85_aftertreatment_, *P* = 0.054); visual learning (36.09 ± 3.12_beforetreatment_ vs. 39.11 ± 1.00_aftertreatment_, *P* = 0.050); reasoning (39.24 ± 3.38_beforetreatment_ vs. 40.00 ± 1.52_aftertreatment_, *P* = 0.056); social recognition (47.15 ± 3.69_beforetreatment_ vs. 49.00 ± 0.79_aftertreatment_, *P* = 0.048); composite (31.26 ± 3.09_beforetreatment_ vs. 30.99 ± 0.28_aftertreatment_, *P* = 0.067). These data demonstrated that Li mono-therapy treatment depressive-phase of BP did not deteriorate the cognitive performance. However, among the groups of DP/Li+LTG, DP/LTG, DP/LTG+VPA groups, the alterations of the elements of the MCCB before and after treatment demonstrated significantly differences, the cognitive performance after treatment, the score of most of the elements decreased; these information demonstrated that LTG, VPA, although they can alleviated the depressive symptoms, they deteriorate the patients cognitive performance. Simultaneously, our data demonstrated that Li mono-therapy in treating the hypo-manic phase of the BP also did not influence the cognitive performance; contrast, VPA demonstrated the deteriorate effect in the cognitive performance (Table 1). Cognitive performance differed among treatment groups. In the DP/Li and MA/Li groups, pre- and post-treatment MCCB scores did not differ significantly (Table 1, indicating that lithium monotherapy did not impact cognitive performance. In contrast, pre- and post-treatment MCCB scores differed significantly in the DP/Li+LTG, DP/LTG, and DP/LTG+VPA groups; most scores were lower after treatment relative to baseline (Table 1), suggesting that LTG and VPA as treatments for the depressive phase of BP impact patients' cognitive performance. In addition, the use of VPA to treat the hypomanic phase of BP worsened patients' cognitive performance (Table 1). Cognitive performance had worsened significantly after treatment in the DP/LTG, DP/Li+LTG, and DP/VPA+LTG groups, with the worst effect observed in the DP/VPA+LTG group. No worsening of cognitive impairment was observed in the DP/Li group (Figures 6A–H). Similarly, cognitive impairment had worsened significantly after treatment in the MA/VPA, but not MA/Li, group (Figures 6I–P).

## Discussion

In this study, drug-naïve patients with BP who experienced their first episodes of depression or hypomania were treated with lithium, alone or in combination with other medications. The results showed that lithium, alone or in combination with LTG, had bidirectional (antidepressant and antimanic) effects through the regulation of depression- and mania-related brain functional abnormalities (reflected by the rs-CBF). In addition, lithium monotherapy did not worsen patients' cognitive performance, as did the other tested treatments. Thus, lithium monotherapy is overall a better treatment option for patients with BP.

Our findings support the antidepressant effect of lithium monotherapy, and the superior antidepressant effect of combination Li+LTG treatment than of monotherapy with either drug, as reflected by rs-CBF data from many brain regions associated with depression. Unexpectedly, LTG monotherapy did not have the same regional effects as did the combination therapy, suggesting the superimposition of lithium's effects to achieve symptom improvement with the combination treatment. The mechanism underlying this effect remains to be elucidated.

VPA and LTG, as monotherapies and as VPA+LTG and Li+LTG, worsened the cognitive impairment of patients with BP in this study. The cognitive side effects of VPA are controversial; observed effects range from protective to the impairment of cognitive ability in patients with epilepsy (54–57). However, the children of women whose mothers were exposed to VPA during pregnancy were found to be at greater risk of cognitive and even IQ impairment (54–57). Our data demonstrated that LTG and VPA both worsened the cognitive impairment of patients with BP, which were inconsistent with previous studies. A future cohort study is needed to further clarify it. Our data demonstrated the beneficial effect of lithium on cognitive function, but this treatment cannot restore cognitive function completely to the status at the time of BP onset. Further research is needed to identify a safe BP treatment that more effectively improves cognitive impairment and alleviates depressive symptoms.

This study has several limitations. First, previous studies reported that the definition of mood stabilizer was: (1) benefits at least one primary aspect of bipolar illness (mania, depression, cycling frequency, number of episodes or sub-threshold symptoms), (2) is effective in both the acute and maintenance phases of treatment, and (3) does not worsen any aspect of the illness. The first generation of mood stabilizers included Lithium, valproate, and carbamazepine. The second generation of mood stabilizer included Lamotrigine, and atypical neuroleptics included Clozapine, Olanzapine, Quetiapine, Aripiprazole, and Risperidone (57–60). More notably, previous studies reported that Quetiapine exert a balance of anti-manic and antidepressant effectiveness (57–60) and had the effect of deteriorate the cognitive impairments in the patients with BP (57–60). Based on this background, Quetiapine should be given priority to be adopted in the present study. However, due to the knowledge insufficiency and condition limited, in present study, Quetiapine and other second generation mood stabilizers did not adopted in this study, this is a pivotal limitation of this study, in the future study, we will adopted the second generation mood stabilizers (especially Quetiapine) to test the priority of them in improving the prognostic of the patients with BP. Second, the sample was small and exclusively male, which introduced selection bias. Further investigation with larger samples of men and women is warranted to better characterize the pathological features of BP and treatment effects. Third, as patients with severe mania cannot comply with the requirements of MRI examination, only hypomanic patients enrolled in this study; this approach may have resulted in sample bias. Future studies should be conducted with patients in moderate to severe manic phases of BP. Fourth, the influence of patient age was regressed in our analysis, but this approach cannot fully eliminate this influence; hence, we will consider the influence of patient age in future work. Fifth, combined treatment with VPA and antidepressant agents may have a greater influence than either monotherapy alone; although we statistically minimized the influence of this factor, it could not be eliminated fully with the methods we used. In future studies, this influence should be addressed using more advanced statistical methods. Sixth, in the present study, all the patients in the present study accepted a fixed dosage of lithium, this may be influence the treatment effect, in the future study, we will conducted a multiple arms study to test the dosage associated treatment effect.

## Conclusion

Taken together, to the best of our knowledge, this study may be the first study using ASL techniques to test the brain blood alterations associated with the treatment effect and cognitive performance of first episode and drug naïve BP both in depressive phase and hypomanic phase. Although with several limitations, our data and previous studies all converged to support that lithium, alone or in combination with other medications can bidirectionally regulates depression- and mania-related brain functional abnormalities (as demonstrated by rs-CBF alterations) in patients with BP. Lithium monotherapy had a better antimanic effect than VPA, was superior to the other tested treatments in improving cognition during the course of BP, and had satisfactory antidepressant effects, albeit slightly lesser than did combined lithium and LTG treatment.

## Data availability statement

The original contributions presented in the study are included in the article/supplementary files, further inquiries can be directed to the corresponding authors.

## Ethics statement

The studies involving human participants were reviewed and approved by the Ethics Committee of Tianjin Fourth Center Hospital. The patients/participants provided their written informed consent to participate in this study.

## Author contributions

CZ, HT, CH, and GC contributed to the conception and writing of the manuscript. XM, JC, QL, LY, RL, QZ, and XS contributed to the article and approved the submitted version. All authors contributed to the article and approved the submitted version.

## Funding

This work was supported by grants from the Tianjin Health Bureau Foundation (2014KR02), National Natural Science Foundation of China (81871052 and 81701326), and the Key Projects of the Natural Science Foundation of Tianjin, China (17JCZDJC35700).

## Conflict of interest

The authors declare that the research was conducted in the absence of any commercial or financial relationships that could be construed as a potential conflict of interest.

## Publisher's note

All claims expressed in this article are solely those of the authors and do not necessarily represent those of their affiliated organizations, or those of the publisher, the editors and the reviewers. Any product that may be evaluated in this article, or claim that may be made by its manufacturer, is not guaranteed or endorsed by the publisher.
